# Case Report: Mediastinal Mass in SARS-COV-2 Pandemic: A Word of Caution

**DOI:** 10.3389/fsurg.2021.648759

**Published:** 2021-03-01

**Authors:** Alessandro Baisi, Alessandra Mazzucco, Giovanni Caffarena, Gerardo Cioffi, Angelo Guttadauro, Ugo Cioffi

**Affiliations:** ^1^Thoracic Surgery Unit, University of Milan-ASST Santi Paolo e Carlo, Milan, Italy; ^2^Department of Sciences and Technologies, University of Sannio, Benevento, Italy; ^3^Department of Surgery, Istituti Clinici Zucchi, University of Milan Bicocca, Monza, Italy; ^4^Department of Surgery, University of Milan, Milan, Italy

**Keywords:** SARS-CoV-2, mediastinal mass, viral infection, lymphoproliferative disease, biopsy

## Abstract

**Background:** SARS-CoV-2 is a new disease with some manifestations not yet well-known. Sharing experiences in this topic is crucial for the optimal management of the patients.

**Case Presentation:** Left upper extremity deep vein thrombosis (UEDVT) due to a mediastinal mass strongly suspected of lymphoproliferative disease in a patient affected by SARS-CoV-2, disappearing at the resolution of the viral infection.

**Conclusion:** Before proceeding to surgical biopsy, mediastinal mass in SARS-CoV-2 patients must be revaluated after the resolution of the infection.

## Introduction

In order to contribute positively to the development of a precise decision-making process, sharing the experiences and perspectives is crucial. We report the case of a positive SARS-CoV-2 female patient candidate to surgical biopsy of a Positron Emission Tomography (PET-TC) positive mediastinal mass which, following the resolution of the viral infection, underwent a complete regression.

## Case Presentation

**Figure 1:**
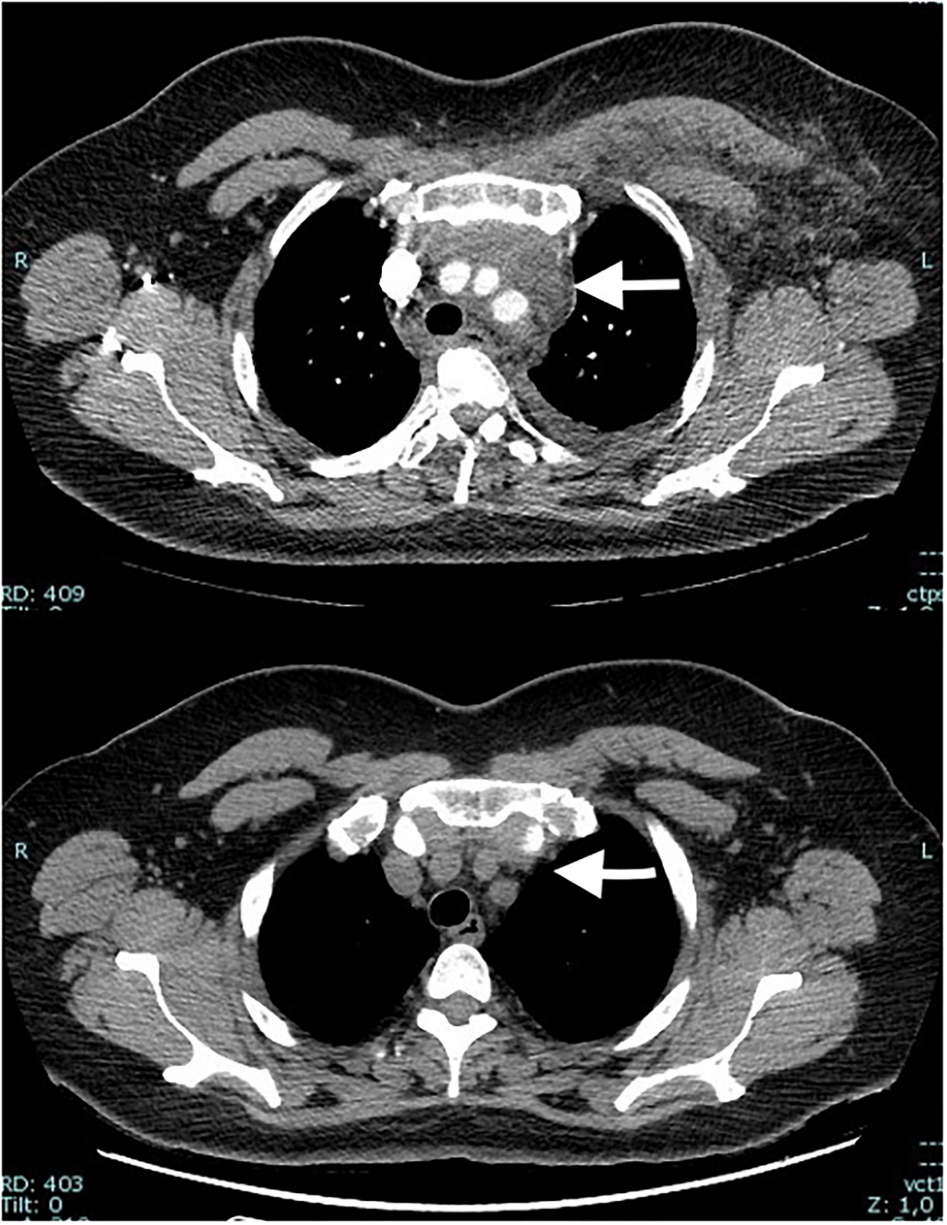
Contrast medium Chest Computed Tomography showing a mass of the upper anterior mediastinum compressing and displacing the left anonymous vein that is occluded (above) (white arrow). After 6 weeks, after the resolution of the SARS-CoV-2 the mass disappeared (white arrow).

**Figure 2:**
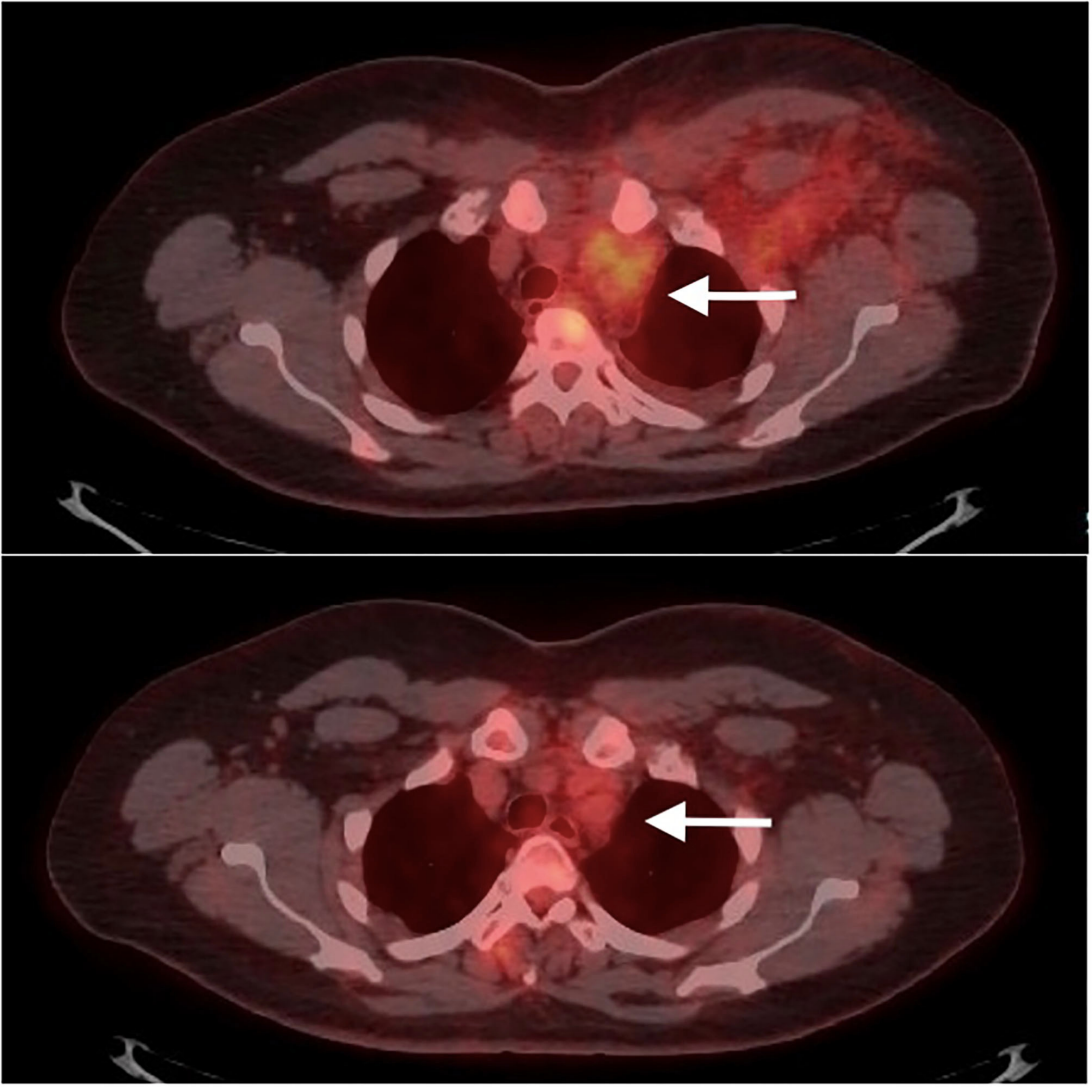
The mediastinal positive PET-CT scan mediastinal mass (above) disappeared after 46 days (below), at the resolution of the SARS-CoV-2.

A 50-year-old woman, former smoker with a negative medical history, went to the emergency room in March 2020 for pain in the neck and left arm. No dyspnea or cough were reported. At clinical examination the upper left arm and the neck showed swelling edema and redness. Blood tests showed leukocytosis, C-reactive Protein and D-dimer elevated. SARS-CoV-2 and H1N1 nasal swabs were negative. An eco-color-doppler showed left upper extremity deep vein thrombosis (UEDVT). The anatomical extension of the thrombosis was up to the left axillary vein, left subclavian vein, left jugular vein. The patient was therefore treated with Low Molecular Weight Heparin (Enoxaparin) 8000 IU BID. The UEDVT was due to a mass in the upper anterior mediastinum compressing and displacing the left anonymous vein evident at a contrast medium Chest Computed Tomography (CT) (Figure 1). Pulmonary Embolisms (PE) of the Right Lower Lobe (RLL) and Left Upper Lobe (LUL) were also present. At a PET-CT (Figure 2) an intense uptake of the mediastinal mass was evident suggesting a lymphoproliferative disease or lymph nodes' metastasis. An ultrasound-guided left supraclavicular lymph node biopsy was performed with negative culture and histological results. A surgical mediastinal mass biopsy by VATS was therefore planned, but a new SARS-CoV-2 nasal swab was positive. This, together to the pulmonary embolism, prompted us to delay the procedure. The patient was relocated in a dedicated SARS-CoV-2 department where she was treated with azithromycin and hydroxychloroquine for 10 days and long-term anticoagulation therapy. The patient was discharged in home isolation protocol 24 days after access (11 from positivity). After the execution of 2 negative SARS-CoV-2 control nasal swabs, respectively, 15 and 20 days after discharge, the patient was revaluated for the surgical procedure. Because the previous PET-CT dated back a couple of months before it was repeated. A complete regression of the mediastinal lesion was evident, confirmed also by a contrast medium CT, that showed also a resolution of the UEDVT.

## Discussion

According to a WHO report on SARS-CoV-2, the disease has no specific manifestation, and the presentation can range from completely asymptomatic to severe pneumonia and death (1). In our patient, the presenting symptom of the SARS-CoV-2 was a UEDVT due to a mass in the anterior mediastinum compressing and displacing the left anonymous vein. There was also some pulmonary embolism due to the well-known thrombotic complication of the disease (2, 3). Interestingly, the mediastinal mass, that was initially strongly suspected of lymphoproliferative disease, disappeared after 2 months with the resolution of the SARS-CoV-2 without any specific therapy. It must therefore be considered as enormous mediastinal confluent lymphadenopathy (4, 5).

Our experience suggests that in SARS-CoV-2 patients affected by mediastinal mass, the evaluation of the mass must be repeated after the resolution of the disease, before proceeding to surgical biopsy.

## Data Availability Statement

The original contributions presented in the study are included in the article/supplementary material, further inquiries can be directed to the corresponding author/s.

## Ethics Statement

Ethical review and approval was not required for the study on human participants in accordance with the local legislation and institutional requirements. The patients/participants provided their written informed consent to participate in this study.

## Author Contributions

All authors participated equally in the case and in the writing of the manuscript.

## Conflict of Interest

The authors declare that the research was conducted in the absence of any commercial or financial relationships that could be construed as a potential conflict of interest.
